# Simultaneous circulation of genotypes I and III of dengue virus 3 in Colombia

**DOI:** 10.1186/1743-422X-5-101

**Published:** 2008-09-02

**Authors:** Jose A Usme-Ciro, Jairo A Mendez, Antonio Tenorio, Gloria J Rey, Cristina Domingo, Juan C Gallego-Gomez

**Affiliations:** 1 Viral Biology – PECET, Sede de Investigación Universitaria, Universidad de Antioquia, A.A. 1226, Medellín, Colombia; 2 Grupo de Inmunovirología, Sede de Investigación Universitaria, Universidad de Antioquia, A.A.1226, Medellín, Colombia; 3 Laboratorio de Virología, Instituto Nacional de Salud, Avenida calle 26 No. 51-20, Bogotá D.C., Colombia; 4 Laboratorio de Arbovirus y Enfermedades Víricas Importadas, Centro Nacional de Microbiología, Instituto de Salud Carlos III, Carretera Majadahonda-Pozuelo Km 2, Majadahonda (28220), Madrid, Spain

## Abstract

**Background:**

Dengue is a major health problem in tropical and subtropical regions. In Colombia, dengue viruses (DENV) cause about 50,000 cases annually, 10% of which involve Dengue Haemorrhagic Fever/Dengue Shock Syndrome. The picture is similar in other surrounding countries in the Americas, with recent outbreaks of severe disease, mostly associated with DENV serotype 3, strains of the Indian genotype, introduced into the Americas in 1994.

**Results:**

The analysis of the 3'end (224 bp) of the envelope gene from 32 DENV-3 strains recently recovered in Colombia confirms the circulation of the Indian genotype, and surprisingly the co-circulation of an Asian-Pacific genotype only recently described in the Americas.

**Conclusion:**

These results have important implications for epidemiology and surveillance of DENV infection in Central and South America. Molecular surveillance of the DENV genotypes infecting humans could be a very valuable tool for controlling/mitigating the impact of the DENV infection.

## Background

Dengue viruses (DENV) belong to the genus *Flavivirus*, transmitted by *Aedes *mosquitoes and constitutes a major concern in public health, infecting millions of people per year in tropical and subtropical areas throughout the world. DENV causes a wide spectrum of clinical manifestations in humans, ranging from a flu-like illness, known as Dengue Fever (DF), to the more severe Dengue Haemorrhagic Fever (DHF) and Dengue Shock Syndrome (DSS).

DENV are enveloped viruses with a positive sense ssRNA of about 11 kb coding a single open reading frame for three structural and seven non-structural proteins [1]. Additionally, DENV comprises four distinct serotypes (DENV-1, DENV-2, DENV-3 and DENV-4) and infection with any of them can produce the most severe manifestations of illness [2].

Although four DENV serotypes can be differentiated by immunofluorescence, it does not provide information about epidemiologic origin and phylogenetic relationship between strains from different geographic regions. In fact, studies of evolution and molecular epidemiology of DENV have demonstrated the occurrence of genotype clusters within each serotype [3-9]. For this reason, genetic characterization of DENV has become a critical issue for understanding epidemic patterns of viral spread. The increase in virus transmission over the last 50 years has possibly increased its adaptive potential, resulting in more virulent genotypes which could be associated with DHF/DSS [10,11].

In Colombia, the four serotypes of DENV have been involved in epidemics, although DENV-1 and DENV-2 have had the higher circulation rate since 1971. Moreover, since the time when the first case of DHF was described, at the end of 1989, these two serotypes have been particularly associated with severe disease. DENV-4 was first detected in 1984 and since then has been sporadically isolated from mild cases of DF.

On the other hand, DENV-3 was detected in Colombia for a short time in 1975 and was then thought to have disappeared from the country [12]. Nevertheless, DENV-3 re-appeared in Latin America in 1994 in Panama [13], and over the next six years rapidly spread to Central, South America and Caribbean countries, causing outbreaks of DF, particularly in Nicaragua, Mexico, Ecuador and Venezuela . DENV-3 was first reported in Venezuela in 1999, and was subsequently detected in Peru and Ecuador in 2000 and Brazil in 2001. In Colombia, 24 years after it had disappeared, DENV-3 was again detected in the state of Santander in 2001 [14], and officially reported by National Health Institute (Instituto Nacional de Salud, INS, Bogotá, Colombia) in early 2002 in state of La Guajira. It then dispersed all over the country, especially in those areas where dengue is endemic. Between 2003 and 2005, DENV-3 was the most frequent serotype reported by the INS. By the year 2006, co-circulation of DENV-1, DENV-2 and DENV-3 was increasingly being detected, particularly in endemic areas (Mendez JA, unpublished data).

In order to determine the arrival and dispersal patterns of DENV-3 in Colombia, a molecular phylogenetic analysis was done using the 3' region of the envelope (*E*) gene from 32 isolates, showing circulation of genotype III, in agreement with previous reports from neighbouring countries [10,15-17]. Additionally, the data shown here support the detection of genotype I, coincident with genotype III. These findings are in accordance with the spatial and temporal co-circulation of distinct genotypes, which could have important implications for the epidemiology of the disease.

## Results and Discussion

### Phylogenetic reconstruction of DENV-3

**Figure 1:**
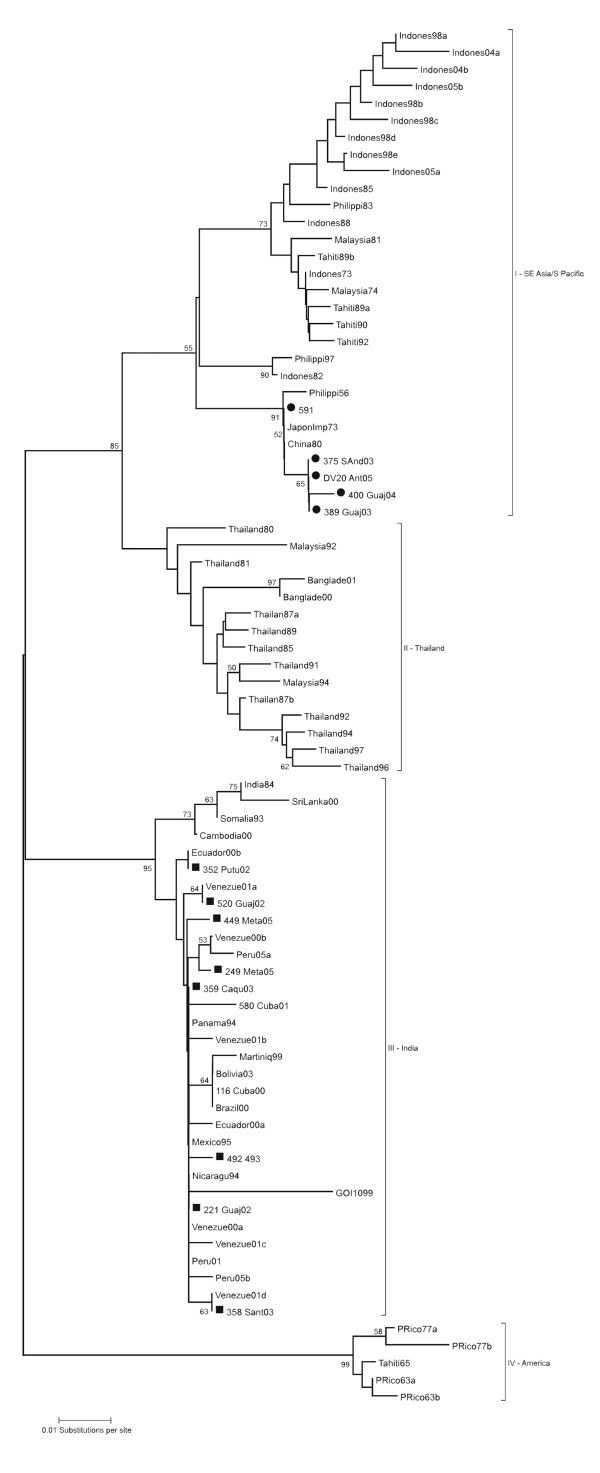
**Neighbor-joining phylogenetic tree of DENV-3 using a 224 bp fragment of the *E *gene**. This figure is showing the presence of two different lineages of DENV-3 in Colombia. The Tamura-Nei nucleotide substitution model was used to estimate distance matrix. Sequences obtained in present study marked with circles and boxes correspond to genotype I and III, respectively. Bootstrap values major of 50% were maintained in the tree supporting clustering in genotypes. Horizontal branch lengths are drawn to scale.

**Figure 2:**
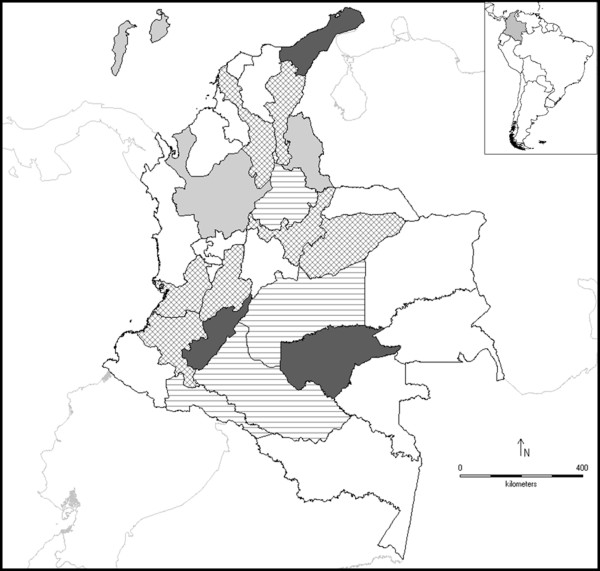
**Distribution of DENV-3 genotypes I and III in Colombia**. Light gray represents the presence of genotypes I, horizontal lines represent the presence of genotype III, dark gray represents the co-circulation of the genotypes I and III; and crossed lines represent DENV-3 occurrence without genotype determination in the present study. The co-circulation of both genotypes is registered in three states of Colombia (La Guajira, Guaviare and Huila).

As shown in the phylogenetic tree (Figure 1), in this study DENV-3 circulation in Colombia was detected since the beginning of 2002. The results were consistent between distance and character-based methods, with minimal differences in topologies (Figure 1, Additional file 1, and data not shown). The most important findings are the detection of genotype I (or Southeast Asia/South Pacific genotype) in Colombia and its co-circulation with genotype III (or Indian genotype) [6,18] in three states from Colombia, La Guajira, Guaviare and Huila (Figure 2). Genetic diversity within 3'end of the *E *gene of DENV-3 throughout the world allowed resolution of previous clustering in four lineages (genotypes) [6], and the presence of a basal clade in genotype I, would be consistent with a fifth genotype [19].

### Genetic diversity within DENV-3

Diversity within DENV-3 has been previously identified and classified [20], but they have found that genetic distance between genotypic groups is low when compared to genetic diversity in DENV-1 and -2, showing that the fixation rate is also lower [18]. By contrast, it has been published that DENV-3 has the higher substitution rate between the dengue viruses (about 7,48 substitutions/site/year) [21]. Our results shows that overall mean distance for DENV-3 as estimated for 84 sequences of 224 bp, with MEGA software is 0,070; for 104 DENV-1 sequences is 0,065 and for 60 DENV-4 sequences is 0,053. Overall mean distance for DENV-2 has not been determined in this study.

### Molecular epidemiology of DENV-3 in Colombia

In the Americas, DENV-3 circulation was reported in the 1960's and 1970's, and all sequenced strains were clustered within genotype IV or American genotype [6,18]. After these isolations, genotype IV has not been identified in any country and could be considered as an extinct genotype. In Colombia, circulation of DENV-3 was reported from 1975 – 1977 [12]. The identification was made by viral isolation in mosquito cells (C6/36) and indirect immunofluorescence, but molecular detection was not carried out. Therefore, sequences of isolated strains during this period have not been determined. It is highly probable that Colombian isolates from this period would cluster within genotype IV, like Puerto Rico strains isolated in the same year [GenBank: L11434].

In the present study, we attempted to amplify historical Colombian strains of DENV-3 isolated in 1977, but it could not be achieved, maybe due to poor samples, or improper maintenance or storage during this time. The recovery of these samples could enrich the basal clade of genotype IV, or might help in explaining the presence of an Asian genotype (genotype I) in Colombia at present if it had been circulating in the past, a very difficult hypothesis to corroborate.

The genomic region used to analyze the relation between strains has been evaluated and determined to be an informative region for genotyping [22]. Nevertheless, the complete *E *gene of some strains has been sequenced, and the topology results are newly confirmed (Additional file 1).

Since DENV-3 genotype III has been present in northeastern and southwestern Colombia since early 2002, different routes of introduction are possible. First, The Venezuelan origin is supported by high similarity of sequences and circulation of this genotype in Venezuela in August of 2001, when the largest epidemic caused by DENV there since the 1989 DENV-2 epidemic ended [17]. The Venezuelan origin of Colombian strains is also supported by the first isolation in La Guajira, along the frontier with Venezuela. Second, it is possible that DENV-3 genotype III had come across the frontier with Peru and/or Ecuador. The high similarity between a strain from Ecuador (DQ177898_Ecuador00) and a strain from Putumayo, Colombia (352_Putu02), a state along the border, offers hard support for this idea. Finally, the entry of genotype III into the Americas was first reported in Panama and Nicaragua in 1994 [13], so another possibility is its introduction into Colombia through the northwestern border with Panama. However, results do not support this hypothesis, not only due to the genetic distance between strains of Colombia and Panama but also mainly due to distribution of strains on northeast and southwest of Colombia. Surely not only one introduction event had occurred, but probably at least two events, via northeast and southwest of Colombia.

DENV-3 genotype I was recently described in the Americas from nine cases in Brazil, as a result of phylogenetic analysis using two fragments corresponding to *C-prM *and partially the *E *gene [23]. Here, we report the presence of this lineage in Colombia from a different region of the *E *gene, without recent closely-related sequences available on GenBank to date. Moreover, the related sequences corresponding to Asian strains were isolated in 1973 in Japan as an imported case and in 1980 in Guangxi, China (GenBank: AB111085 and AF317645). Samples that clustered in this lineage are located in a basal branch into genotype I, with high bootstrap support (86%) and mean distance between clades of 5%, estimated with Tamura-Nei model to be classified as a fifth different genotype, referred to as genotype V in [19]. Variability within genotype I has been demonstrated as the presence of into-clade nucleotide substitutions and branching in few years (Figure 1).

The presence of DENV-3 genotype I only in Colombia, and its close relation with Asiatic strains from 1973 and 1980, suggests that strains circulating in Colombia during the 1970's would have not been of genotype IV, like other American strains from that period, but, perhaps a strain of Asiatic origin that had been circulating without detection for over 25 years until 2002. This speculation needs more data to support it, because there is no evidence for genotype circulation in Colombia in the past, and explaining possible silent circulation without causing outbreaks for more than twenty years could be a challenge.

The presence of the Southeast Asia/South Pacific genotype has recently been detected not only in Colombia, but also in Brazil [23].

DENV-3 genotype IV was last reported in Puerto Rico in 1977 (as corroborated by sequencing) [24], but to date Colombian isolates from the same year have not been sequenced because of lack of good samples from these years. Reintroduction of other genotypes clearly has not signified displacement of genotype IV, probably because it was not present for more than twenty years, so co-circulation was not possible.

Intra-serotype recombination has been detected in natural populations of DENV [22,25-29]. Nonetheless, the significance of recombination events for increasing genetic diversity is unknown. The topology of the phylogenetic tree could be affected by recombination between strains, and then the results could be misinterpreted. Our findings obtained by using a short fragment could be a product of recombination. For this reason, we achieved sequencing of complete *E *gene of strains corresponding to both DENV-3 Colombian genotypes. The results of the phylogenetic reconstruction (Additional file 1) were consistent with the presence of genotypes III and I (genotype V according to [19]). Additionally, a recombination analysis using the complete *E *gene was carried out, but recombination events were not detected (data not shown).

As known, the potential for causing severe disease has been described for all four serotypes of DENV, and the main factors considered to explain its pathogenicity are host genetic susceptibility, antibody dependent enhancement and differences in virulence among strains [30]. It is evident that phenotype is not segregating with phylogeny, but is an evolutionary convergence, resulting from interaction of the viruses with hosts and moulded by selection to enhance its transmission and persistence [31].

Determinants of virulence have been located in three genomic regions [32] and have been tested *in vitro *[33], so the genotypes have been more or less related with potential to cause DHF. Recent studies have concluded that the spread of genotype III of DENV-3 from the Indian subcontinent to Africa and then to Latin America was correlated with an increase in severe cases of dengue disease [10,34]. The ability of all serotypes to cause severe disease is an indicator of adaptive selection of this character during independent evolution of DENV serotypes. However, more efforts should be made to understand the role of viral genetics in human pathogenesis.

Although the origin of genotype I is uncertain, the co-circulation with genotype III could have epidemiologic implications if it has intra-serotype antigenic variation related with differential generation of protective antibodies and immune response [6]. It is important to take into account the low sample size, because the possibility of a more wide distribution of the genotypes I and III into the country (Figure 2).

The relevance of these results is the detection of two different genotypes in the same country, one of them of Asiatic origin, only recently described in the Americas [23]. The results underscore the need for a global strategy of genotypes circulation surveillance, because disease dynamic is more than a regional problem, involving neighbouring countries as well. The establishment of a Pan-American program would provide very useful epidemiological information about the potential of strains for causing outbreaks.

## Methods

### Clinical samples

The strains included in the study, with locality, year and GenBank accession numbers, are listed in table 1. Samples were collected by local hospitals in Medellín (Colombia) and Public Health Laboratories of the National Network all around the country and remitted to the National Institute of Health (Colombia) for diagnostic and epidemiological surveillance. Serum or plasma was obtained and kept at -70°C until processing. The samples cover a period of four years since reintroduction and detection of DENV-3 in Colombia (2002 – 2005).

**Table 1:** Colombian strains of DENV-3 sequenced in the present study.

Strain*	**Name**	**Location**	**Date**	**Genbank accession #**	**Genotype (Subtype)**
388280	375_SAnd03	San Andrés	11/09/2003	EU003494	SE Asia/S.Pacific (I)
388887	389_Guaj03	Guajira	14/11/2003	EU003495	SE Asia/S.Pacific (I)
389520	395_NSan04	Norte de Santander	20/01/2004	EU003496	SE Asia/S.Pacific (I)
390192	400_Guaj04	Guajira	09/02/2004	EU003497	SE Asia/S.Pacific (I)
391300	417_Guav04	Guaviare	15/07/2004	EU003498	SE Asia/S.Pacific (I)
391933	429_Huil04	Huila	15/10/2004	EU003499	SE Asia/S.Pacific (I)
V-599	591VI	-	-	EU003511	SE Asia/S.Pacific (I)
-	DV06_Ant05	Antioquia	22/06/2005	EU003514	SE Asia/S.Pacific (I)
-	DV20_Ant05	Antioquia	21/11/2005	EU003513	SE Asia/S.Pacific (I)
384119	520_Guaj02	Guajira	22/01/2002	EU003509	India (III)
384584	221_Guaj02	Guajira	27/03/2002	EU003483	India (III)
384826	484_Putu02	Putumayo	11/04/2002	EU003504	India (III)
385233	352_Putu02	Putumayo	04/06/2002	EU003487	India (III)
386891	517_Caqu03	Caquetá	14/03/2003	EU003507	India (III)
386990	358_Sant03	Santander	01/04/2003	EU003488	India (III)
387023	359_Caqu03	Caquetá	04/04/2003	EU003489	India (III)
387124	363_Caqu03	Caquetá	14/04/2003	EU003490	India (III)
387129	366_Caqu03	Caquetá	14/04/2003	EU003491	India (III)
387130	367_Caqu03	Caquetá	14/04/2003	EU003492	India (III)
387131	368_Caqu03	Caquetá	14/04/2003	EU003493	India (III)
387173	464_2003	-	24/04/2003	EU003503	India (III)
388446	233_Guaj03	Guajira	22/09/2003	EU003484	India (III)
391713	518_Putu04	Putumayo	22/09/2004	EU003508	India (III)
391771	535_Huil04	Huila	27/09/2004	EU003512	India (III)
392438	530_Guav05	Guaviare	03/02/2005	EU003510	India (III)
393084	449_Meta05	Meta	12/05/2005	EU003500	India (III)
393198	456_Meta05	Meta	26/05/2005	EU003501	India (III)
393273	247_Guav05	Guaviare	10/06/2005	EU003485	India (III)
393282	249_Meta05	Meta	10/06/2005	EU003486	India (III)
393492	461Guav05	Guaviare	07/07/2005	EU003502	India (III)
469-1	492VI	-	-	EU003505	India (III)
470-12	493VI	-	-	EU003506	India (III)

*Code in Laboratorio de Virologia, INS repository (Instituto Nacional de Salud, Bogotá, Colombia).

### Virus isolation

C6/36 cells cultured in Dulbecco's modified Eagle's medium (DMEM), were infected with 0.15 ml of samples and incubated for 10 days at 28°C, washed with PBS, removed by hitting the culture tubes manually and seeded on slides. Cells were then fixed with acetone and the indirect immunofluorescence procedure was carried out incubating the cells with serotype-specific monoclonal antibodies (kindly donated by Dr. Elizabeth Hunsperger, CDC Puerto Rico) for 60 minutes and then washed with PBS and incubated for another 60 minutes with a commercial secondary antibody conjugated with fluorescein isotyocianate.

### RNA extraction

Aliquots of 140 μl of serum or supernatants of cell cultures were placed into 540 μl of AVL buffer with Carrier RNA and used to extract the viral RNA with QIAamp Viral RNA Minikit (QIAGEN, Germany) as indicated by manufacturer. RNA obtained in 60 μl of AVE buffer was stored at -70°C and used in the RT-PCR. Alternatively, the total RNA of some samples was extracted by the use of TRIZOL^® ^LS (INVITROGEN, Inc., USA), and a final volume of 15 μl was recovered in these cases.

### RT-PCR and nested-PCR

The RT-PCR and nested-PCR have been previously described [35]. When viral load was too low, nested-PCR was used to detect DENV directly on clinical samples, so sensitivity of detection was increased more than five logarithms and passage of viruses in cell cultures was avoided (data not shown). RT-PCR primers were designated to amplify an intergenic region *E/NS1 *of 776 bp, and nested-PCR to amplify an internal region of 350 bp.

### DNA sequencing

Products of RT-PCR or nested-PCR were purified using QIAquick PCR Purification Kit (QIAGEN, Germany). Sequencing reactions on both strands were performed with 10 pmol of the primers used for the second round of amplification, and the ABI Prism Dye Terminator Cycle Sequencing Ready Reaction Kit (APPLIED BIOSYSTEMS, USA), and analysed using an ABI model 377 automated sequencer (APPLIED BIOSYSTEMS, USA).

### Sequence editing

Four sequences were obtained for each sample, two sequences with sense and two with antisense primer. Editing and consensus obtaining were performed with the SeqMan module of Lasergene (DNASTAR Inc. Software, Madison, Wis.).

Sequences on GenBank corresponding to different lineages of DENV-3 were downloaded and aligned with the consensus sequences obtained in this study, using Clustal W software [36]. Additionally, a visual correction of alignment was done. A fragment of 224 bp was used for phylogenetic reconstructions corresponding to the 3' end of the *E *gene (nucleotides 1256 to 1479). The portion of the *NS1 *gene amplified with the nested-PCR was excluded from the analysis due to the absence of this portion in the majority of reported sequences.

### Phylogenetic analysis

Alignment of the sequences obtained in the present study (n = 32) (Table 1) and homologous sequences for DENV-3 available on GenBank (n = 68) (Table 2) were used for phylogenetic reconstructions. Many sequences of different strains were completely identical to the fragment analysed, and so one sequence was used for analysis, corresponding to the first isolation.

**Table 2:** List of isolates used in the present study with GenBank accession number, year and location.

**GenBank Accesión #**	**Label**	**Genotype**^a^	**Year**	**Location**
AB189125	Indones98a	I	1998	Indonesia
AY858037	Indones04a	I	2004	Indonesia
AY858043	Indones04b	I	2004	Indonesia
AY858039	Indones98b	I	1998	Indonesia
AY912455	Indones98c	I	1998	Indonesia
AY912454	Indones98d	I	1998	Indonesia
L11428	Indones85	I	1985	Indonesia
AY858038	Indones88	I	1988	Indonesia
L11429	Malaysia74	I	1974	Malaysia
L11425	Indones73	I	1973	Indonesia
AB189128	Indones98e	I	1998	Indonesia
DQ401695	Philippi97	I	1997	Philippines
AB219139	Indones05a	I	2005	Indonesia
AB219138	Indones05b	I	2005	Indonesia
AY744680	Tahiti90	I	1990	Tahiti
L11427	Malaysia81	I	1981	Malaysia
L11619	Tahiti89a	I	1989	Tahiti
AY744678	Tahiti89b	I	1989	Tahiti
DQ401690	Indones82	I	1982	Indonesia
AY744684	Tahiti92	I	1992	Tahiti
L11432	Philippi83	I	1983	Philippines
AF317645	China80	I (V)^b^	1980	China
M93130	Philippi56	I (V)^b^	1956	Philippines
AB111085	JaponImp73	I (V)^b^	1973	Japan
AF147457	Malaysia92	II	1992	Malaysia
AY676370	Thailand81	II	1981	Thailand
AY676368	Thailand85	II	1985	Thailand
AY676359	Thailand80	II	1980	Thailand
AF533079	Thailan87a	II	1987	Thailand
AY135419	Thailan87b	II	1987	Thailand
AY145715	Thailand89	II	1989	Thailand
AY145716	Thailand91	II	1991	Thailand
AY338493	Malaysia94	II	1994	Malaysia
AY145730	Thailand97	II	1997	Thailand
AY145726	Thailand96	II	1996	Thailand
AY145718	Thailand92	II	1992	Thailand
AY145723	Thailand94	II	1994	Thailand
AY496872	Banglade01	II	2001	Bangladesh
AB111080	Banglade00	II	2000	Bangladesh
L11424	India84	III	1984	India
AY099336	SriLanka00	III	2000	SriLanka
AY099337	Martiniq99	III	1999	Martinique
AB111081	Cambodia00	III	2000	Cambodia
AY702032	116_Cuba00	III	2000	Cuba
AY038605	Brazil00	III	2000	Brazil
AY146772	Venezue01a	III	2001	Venezuela
AY146765	Venezue00a	III	2000	Venezuela
AY146767	Venezue00b	III	2000	Venezuela
AY146776	Venezue01b	III	2001	Venezuela
AY702030	580_Cuba01	III	2001	Cuba
AY702033	Nicaragu94	III	1994	Nicaragua
DQ341209	Panama94	III	1994	Panama
DQ341208	Somalia93	III	1993	Somalia
DQ341202	Mexico95	III	1995	Mexico
DQ371245	Venezue01c	III	2001	Venezuela
DQ177899	Ecuador00a	III	2000	Ecuador
DQ177900	Peru01	III	2001	Peru
DQ367720	Venezue01d	III	2001	Venezuela
DQ177898	Ecuador00b	III	2000	Ecuador
DQ177902	Peru05a	III	2005	Peru
DQ177897	Peru05b	III	2005	Peru
DQ177887	Bolivia03	III	2003	Bolivia
AY960630	GOI1099	III	-	Brazil
L11434	PRico77a	IV	1977	Puerto Rico
L11439	Tahiti65	IV	1965	Tahiti
AY146762	PRico63a	IV	1963	Puerto Rico
L11433	PRico63b	IV	1963	Puerto Rico
AY146761	PRico77b	IV	1977	Puerto Rico

^a ^Genotypes as reported by Lanciotti *et al*. (1994).

^b ^Genotype V as reported by Wittke *et al*. (2002).

The strain 359_Caqu03 was completely identical to 363_Caqu03, 366_Caqu03, 367_Caqu03, 368_Caqu03, and 464_2003; strain 449Meta05 was identical to 456_Guav05 and 461Guav05; strain 352_ Putu02 to 484_Putu02; strain 221_Guaj02 to 233_Guaj03, 517_Caqu03, 518_putu04, 247_Guav05 and 530_Guav05; and finally, strain 375_SAnd03 was identical to 389_Guaj03, 395_NSan04, 417_Guav04, 429_Huil04, 535_Huil04 and DV06_Ant05.

The phylogenetic trees were estimated for the 224 bp fragment, corresponding to the 3' end of the *E *gene. Initially, the neighbour-joining algorithm was used with 10000 bootstrap replicates and the Tamura-Nei model of nucleotide substitution with MEGA 3.1 software [37]. Maximum parsimony and Maximum Likelihood trees were obtained with PAUP* [38]. For selecting the model of substitution, MODELTEST software and current dataset were used and the resulting parameters were used for running maximum likelihood analysis. Trees were rooted using genotype IV, only for graphical purposes.

## Conclusion

The more important finding of this work is the co-circulation of genotype III of DENV-3, widely distributed, and the recently reported genotype I, never before described in the Americas, in three Colombian states. Co-circulation of different genotypes in an area could be related with the current association between DENV-3 infection and severity of disease. Moreover, intra-serotype antigenic variation related with differential generation of protective antibodies and immune response could be one of the reasons for the high epidemiological impact of DENV-3 in the Americas.

## Competing interests

The authors declare that they have no competing interests.

## Authors' contributions

JAUC contributed to the experimental design, carried out the experiments and phylogenetic analysis, and drafted the manuscript. JAM contributed to the experimental design, carried out the experiments and provided a critical review of the manuscript. AT conceived the study, its experimental design and provided a critical review of the manuscript. GJR contributed to the experimental design and provided a critical review of the manuscript. CD participated in the experimental design, contributed to the interpretation of data and the critical review of the manuscript. JCGG conceived the study, participated in its design and coordination and finalised the manuscript. All authors read and approved the final version of the manuscript.

## Supplementary Material

Additional file 1 Neighbor-joining phylogenetic tree of the DENV-3 *E *gene corroborating the presence of two different lineages. The Tamura-Nei nucleotide substitution model was used to estimate distance matrix. Sequences obtained in present study marked with circles and boxes correspond to genotype I and III, respectively. Bootstrap values major of 50% were maintained in the tree supporting clustering in genotypes after 1000 pseudo-replications. Horizontal branch lengths are drawn to scale. Click here for file
