# Supplementary figures and images for: Simultaneous circulation of genotypes I and III of dengue virus 3 in Colombia
Source: Virol J. 2008 Sep 2;5:101. doi: 10.1186/1743-422X-5-101 (PMC2553081; doi:10.1186/1743-422X-5-101)

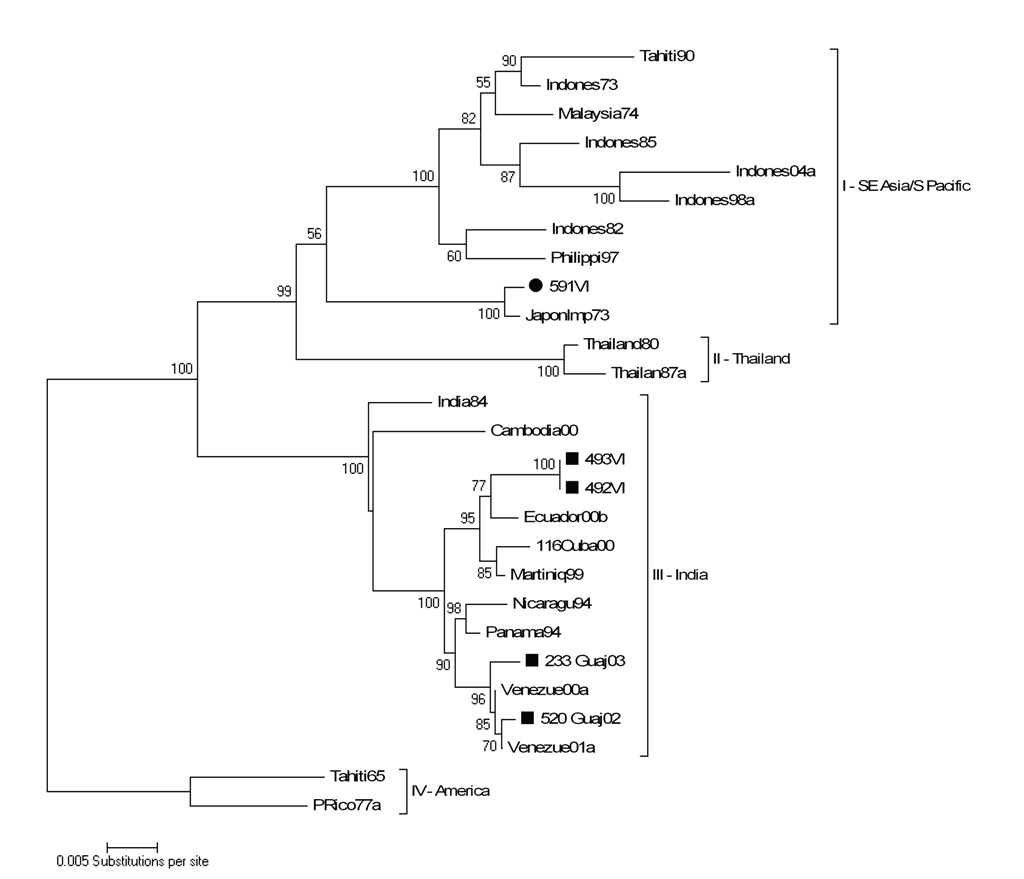

Supplement: Additional file 1 — Neighbor-joining phylogenetic tree of the DENV-3 E gene corroborating the presence of two different lineages. The Tamura-Nei nucleotide substitution model was used to estimate distance matrix. Sequences obtained in present study marked with circles and boxes correspond to genotype I and III, respectively. Bootstrap values major of 50% were maintained in the tree supporting clustering in genotypes after 1000 pseudo-replications. Horizontal branch lengths are drawn to scale. [file 1743-422X-5-101-S1.jpeg]
